# Screening and characterizing of xylanolytic and xylose-fermenting yeasts isolated from the wood-feeding termite, *Reticulitermes chinensis*

**DOI:** 10.1371/journal.pone.0181141

**Published:** 2017-07-13

**Authors:** Sameh Samir Ali, Jian Wu, Rongrong Xie, Feng Zhou, Jianzhong Sun, Miao Huang

**Affiliations:** 1 Biofuels Institute, School of the Environment and Safety Engineering, Jiangsu University, Zhenjiang, China; 2 Botany Department, Faculty of Science, Tanta University, Tanta, Egypt; National Renewable Energy Laboratory, UNITED STATES

## Abstract

The effective fermentation of xylose remains an intractable challenge in bioethanol industry. The relevant xylanase enzyme is also in a high demand from industry for several biotechnological applications that inevitably in recent times led to many efforts for screening some novel microorganisms for better xylanase production and fermentation performance. Recently, it seems that wood-feeding termites can truly be considered as highly efficient natural bioreactors. The highly specialized gut systems of such insects are not yet fully realized, particularly, in xylose fermentation and xylanase production to advance industrial bioethanol technology as well as industrial applications of xylanases. A total of 92 strains from 18 yeast species were successfully isolated and identified from the gut of wood-feeding termite, *Reticulitermes chinensis*. Of these yeasts and strains, seven were identified for new species: *Candida gotoi*, *Candida pseudorhagii*, *Hamamotoa lignophila*, *Meyerozyma guilliermondii*, *Sugiyamaella* sp.1, *Sugiyamaella* sp. 2, and *Sugiyamaella* sp.3. Based on the phylogenetic and phenotypic characterization, the type strain of *C*. *pseudorhagii* sp. nov., which was originally designated strain SSA-1542^T^, was the most frequently occurred yeast from termite gut samples, showed the highly xylanolytic activity as well as D-xylose fermentation. The highest xylanase activity was recorded as 1.73 and 0.98 U/mL with xylan or D-xylose substrate, respectively, from SSA-1542^T^. Among xylanase-producing yeasts, four novel species were identified as D-xylose-fermenting yeasts, where the yeast, *C*. *pseudorhagii* SSA-1542^T^, showed the highest ethanol yield (0.31 g/g), ethanol productivity (0.31 g/L·h), and its fermentation efficiency (60.7%) in 48 h. Clearly, the symbiotic yeasts isolated from termite guts have demonstrated a competitive capability to produce xylanase and ferment xylose, suggesting that the wood-feeding termite gut is a promising reservoir for novel xylanases-producing and xylose-fermenting yeasts that are potentially valued for biorefinery industry.

## Introduction

Wood-feeding termites (order: Isoptera), a gold mine of science and plague of buildings, are the most impressive and effective wood-decomposing systems on the earth in terms of their processing efficiency and scales [1]. Unlike most animals, termites harbor more than 200 symbiotic microbial species that produce an array of cellulosic and hemicellulosic degrading enzymes to digest cellulose and hemicellulose components of the wood. These enzymes, in turn, have a potential value for the bio-ethanol production from lignocellulosic biomass in the biorefinery industries [2]. The termite-gut microbial community usually contains a highly diversified microflora, including various bacteria, yeasts, and protists. The sets of enzymes, co-factors, and genes produced by the termites and their symbiotic consortia are named as the 'digestome' [3]. Within one microliter of the digestome environment, it is possible for the consortia to break down 74–99% of the cellulose and 65–87% of the hemicellulose that they ingest [4] into monosaccharides with 100% of lignocellulose digestion efficiency within this microscale bioreactor [3,5]. Without microbial consortia residing in their gut system, termites would be unable to digest cellulosic biomass. In a previous study, Brune [6] suggested two biological functions of termite-gut consortia as nutrient provision and breakdown of lignocellulose to support the host. In 1923, Cleveland [7] classified termites into two groups, lower and higher termites according to the presence of cellulolytic protozoans in their hindguts. Several studies have further shown an astonishing biodiversity in termite gut symbionts for most wood-feeding lower termites, such as in the genus of *Reticulitermes*; and these gut symbionts are primarily represented by prokaryotes and eukaryotes, particularly including fungi, notably yeasts, and flagellated protozoa being classified in eukaryote category [8].

Termites appear to use their own enzymes synergistically with exogenous enzymes from their gut symbionts for cellulose depolymerization [9]. However, much remains to be learned about such synergism. Hemicellulose-degrading microorganisms as the symbionts and their enzymes also play an important role in the recycling of carbon [8]. These symbiotic microorganisms can effectively produce the xylanases, which have been received much attention for their functions in the hydrolysis of hemicellulose, and further turned hemicellulose to be various value-added products, such as arabinose, glucose, galactose, mannose, and xylose [10]. Xylan is the major abundant hemicellulosic component, formed of D-xylose units, which linked together by β-1,4-glycosidic bonds. It is a unique type of useful fermentation substrates for biofuel industries. Endo-1,4-β-xylanase (EC 3.2.1.8) and β-xylosidase (EC 3.2.1.37) are the major types of xylanases that can be responsible for the hydrolysis of xylan [11]. Due to huge industrial application demands, lots of efforts have been devoted towards bacterial and fungal xylanases as tough competitors in the industrial arena. The xylanases, as one type of important enzymes, are reported with numerous industrial and biotechnological applications, including bioconversion of lignocellulose to fermentable sugars, production of cellophane and rayon, textile industry, production of chemicals such as cellulose ethers and esters, wines clarification, animal feeds, improvement of bread quality, and also in pulping and bleaching processes [12]. However, the high costs of xylanase enzyme production hinder its application in bioethanol production as well as other fields [13]. In addition, very few thermostable xylanases have been reported from thermophilic microorganisms isolated from the gut symbionts of wood-feeding termites [14]. Therefore, to produce xylanases in a large amount, researchers have focused on the screening of new microbial strains from a wide range of microorganisms like fungi, bacteria, and actinomycetes as an alternative to reduce the production costs. A novel yeast species of the genus *Sugiyamaella* was reported with an ability of xylanases production, which was isolated from the gut of the lower termite *Mastotermes darwiniensis* [15]. In addition, a new xylanase-producing Gram-positive bacterium was also isolated from the termite gut, *Reticulitermes santonensis* [16].

During the process of ethanol fermentation, lignocellulosic biomass is conventionally hydrolyzed by acids and the relevant enzyme complexes [17,18]. The C6 sugars are easily fermented to ethanol but xylose (C5), as a major product of the hydrolysis of hemicellulose, is much more difficult to be fermented with regular yeasts. Therefore, an effective fermentation processing on xylose substrate is really important in bioethanol industry, which currently remains an intractable challenge waiting for new solutions. Actually, a number of attempts have been made in the past decade to isolate some stable yeast species that are capable of utilizing xylose. Many known xylose-fermenting yeasts, such as *Candida lignicola*, *C*. *queiroziae*, and *C*. *coipomoensis* are associated with wood-feeding insects, which are able to ferment D-xylose to ethanol [19]. In addition, *Candida shehatae*, *Pachysolen tannophilus*, *Pichia stipitis* and some genetically engineered xylose-fermenting yeasts (including *Saccharomyces cerevisiae*) have also been added in references [20–22]. However, these strains are relatively poor in their performance when applied as the fermenting yeasts in an industry processing [23].

Wood-feeding termites are considered to be the smallest and the most efficient natural bioreactor in the world [4]. The learning from termites and further applying of their gut microbial consortia for wood decomposition and conversion, both from mechanism and microbial resource point of view, in biorefinery industry remains unexplored or at its early stage. Conventionally, most researchers have focused their attention on xylanase-producing filamentous fungi that are considered as the most efficient lignocellulolytic enzyme producers [12], which inevitably leads to, only a limited number of studies on other microbial resources, such as the yeasts resided in the wood-feeding insects [24]. Some symbiotic yeasts from insect guts, such as the genera of *Candida*, *Pichia*, *Rhodotorula*, *Sugiyamaella* and *Wickerhamomyces* [24], are very unique for their functions, which can possibly ferment some C5 or C6 sugars, and simultaneously may produce some key enzyme complexes during the degradation processing of biomass. Those symbiotic yeasts may represent a novel microbial source of hydrolytic enzymes with their unique traits for potential industry values. Therefore, this study was to identify and characterize some novel yeast species or strains hosted in the gut of a wood-feeding termite species, *Reticulitermes chinensis*, where our attention was particularly put on those yeasts that are able to produce xylanolytic enzymes and, simultaneously, also to ferment D-xylose (S1 Fig).

## Materials and methods

### Termite collection and screening of xylanase-producing yeasts

In the present study, the wood-feeding termite, *R*. *chinensis*, was collected from rotting wood trees at Huazhong Agricultural University, Wuhan, China. This site belongs to Hubei Province (31° 12ʹ N 112° 18ʹ E) which characterized by a humid subtropical climate with average temperatures of 1 to 6°C in winters, while summers are hot and humid punishing temperatures of 40°C or above at Wuhan. All necessary permits for termite collections were obtained, and the insects were transported to the Unit of Entomology at Biofuels Institute, Jiangsu University, China. The methods used for isolating yeasts from the termite guts were previously described in details by Suh and Blackwell [25]. From the collected *R*. *chinensis*, 30 individual termite worker samples were transferred to the laboratory of Microbiology of Biofuels Institute at Jiangsu University. The termite guts were removed aseptically and then transferred for crushing with 0.7% sterile saline solution. The crushed solutions were inoculated, separately, in Erlenmeyer flasks with sterile liquid yeast nitrogen base (YNB) containing either D-xylose or xylan according to the method described by Morais et al. [26]. When the yeast growth was detected on the cultured media, aliquots of the cultures (0.5 mL) were then transferred to sterile tubes containing 5 mL YNB-D-xylose or YNB-xylan, and then incubated at 25°C on an orbital shaker at 150 rpm for 3–10 days. By the end of the incubation period, one loopful of each culture was streaked on YNB agar media containing D-xylose or xylan. The inoculated plates were incubated at 25°C until the development of yeast colonies [27]. On yeast extract-malt extract (YM) agar (3 g yeast extract; 3 g malt extract; 10 g glucose; 5 g peptone; 20 g agar and 0.2 g chloramphenicol L^-1^), the different morphotypes were purified by several re-streaking processings. The purified strains were suspended in YM broth supplemented with 50% glycerol and then preserved at -70°C for further identification. A modified plate assay [28] was conducted to evaluate the capacity of xylanase production. All isolates were cultured on xylan-agar medium (YNB, 6.7 g/L; xylan, 10 g/L; pH 5.0) and incubated at 25°C for 5–15 days. Xylanase-producing yeasts were eventually identified based on the formation of clear halo zones around yeast colonies.

### Phenotypic characterization of yeast strains

The morphological, biochemical and physiological traits of the isolated yeasts were first examined by standard methods [19,29]. Carbon assimilation tests were then examined in the liquid media [29]. Starved inocula were also used to conduct the assimilation of nitrogen sources on solid media [30]. In order to assess budding cells, pseudohyphae, and ascospore formation, the yeast strains were cultivated individually or in combinations on YM broth, YM agar and cornmeal agar at 25°C up to 30 days [19].

### DNA sequencing and phylogenetic analyses

**Fig 1:**
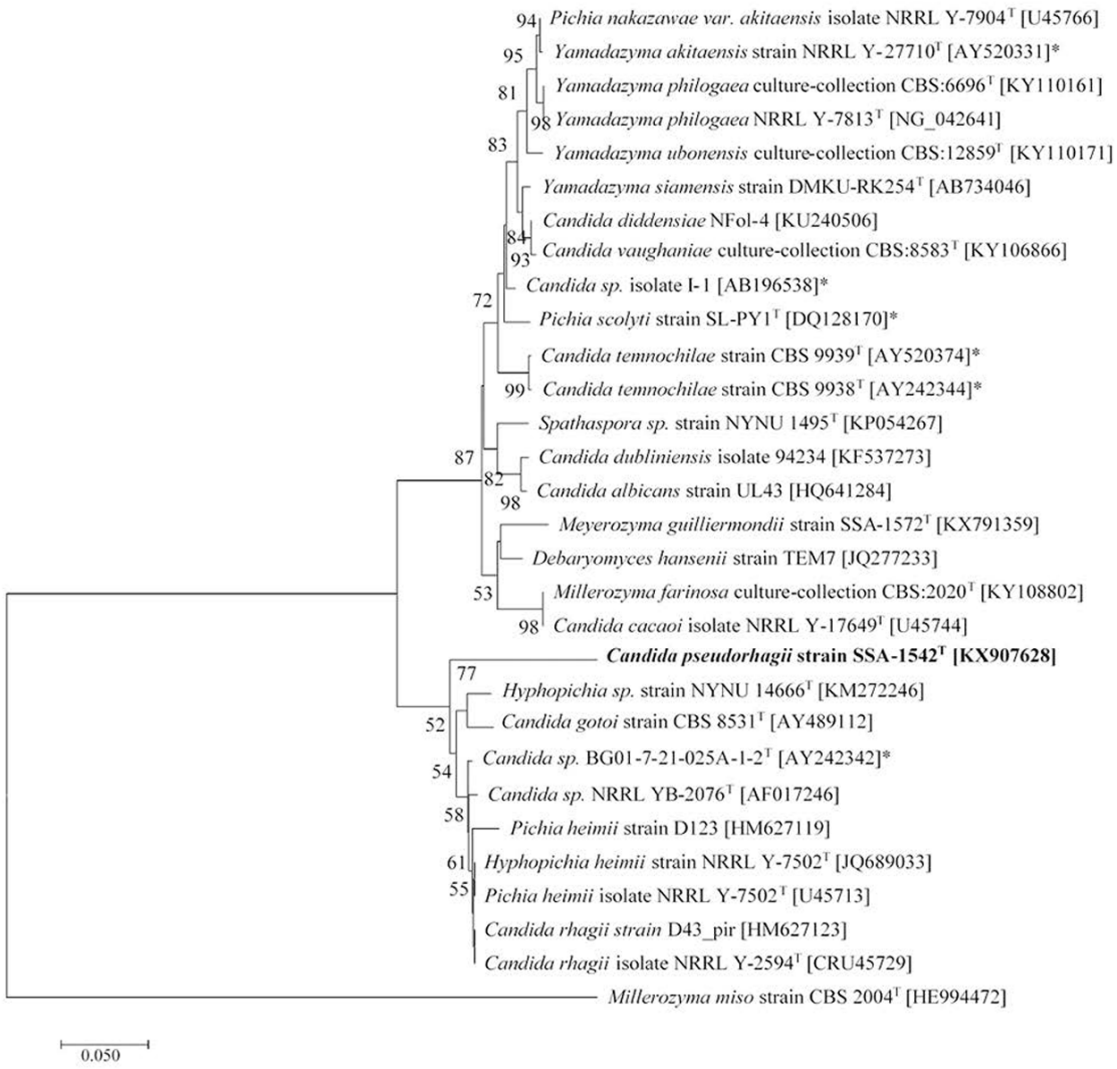
A Neighbor-Joining phylogenetic tree of *C*. *pseudorhagii* sp. nov. strain SSA-1542^T^ with its closely related taxa. The tree was constructed based on the evolutionary distance calculated using Kimura-2 parameter from the nucleotide sequence of D1/D2 domains. The percentage of replicate trees in which the associated taxa clustered together in the bootstrap test (1000 replicates) is shown next to the branches for values ˃ 50%. The bar represents 0.05 substitutions per nucleotide position. GeneBank accession numbers are mentioned within the parentheses. *Millerozyma miso* was an outgroup in the analysis. ^T^ = Type strain. Insect-associated yeasts are marked with asterisks (*).

The methods applied for yeast genomic DNA extraction, PCR amplification, as well as DNA sequencing were actually performed according to an earlier reference [25]. The D1/D2 region of large-subunit rRNA gene and internal transcribed spacer (ITS) region were amplified with primers, NL1/NL4 and ITS1/ITS4, respectively. The purified products were checked by agarose gel electrophoresis, and then submitted to Sangon Biotech (Shanghai, China) for sequencing. The sequences were compared pairwise to those of other related yeast species retrieved from the GeneBank database using BLAST search program [31], and then aligned with the multiple alignment program CLUSTAL X [32]. A phylogenetic tree based on the D1/D2 domains was constructed by using MEGA software version 7.0 [33]. The evolutionary history was inferred using the Neighbor-Joining method [34]. Bootstrap analysis [35] was performed from 1000 replications to determine confidence levels of the clades, and only values ˃ 50% were recorded on the resulting tree. The evolutionary distances were computed using the Maximum Composite Likelihood method [36]. The final dataset contained 456 aligned nucleotide positions. The reference sequences indicated in the tree (Fig 1) and their accession numbers were retrieved from GeneBank database.

### Nomenclature

The electronic version of this article in Portable Document Format (PDF) in a work with an ISSN or ISBN will represent a published work according to the International Code of Nomenclature for algae, fungi, and plants, and hence the new names contained in the electronic publication of a PLOS ONE article are effectively published under that Code from the electronic edition alone, so there is no longer any need to provide printed copies.

In addition, new names contained in this work have been submitted to MycoBank from where they will be made available to the Global Names Index. The unique MycoBank number can be resolved and the associated information viewed through any standard web browser by appending the MycoBank number contained in this publication to the prefix http://www.mycobank.org/MB/. The online version of this work is archived and available from the following digital repositories: PubMed Central; LOCKSS.

### Determination of xylanase activity

The preliminary screening of xylanase-producing strains was later confirmed quantitatively by determining the activity of xylanase production. The positive strains were induced with YNB-D-xylose or YNB-xylan at 30°C, on an orbital shaker at 150 rpm for 3 days and the xylanase activity was then evaluated as described by Morais et al. [26]. The endo-1,4-β-xylanase was also assayed [37]. Briefly, the mixture consisting of 100 μl culture supernatant with 300 μl of a 10 g beechwood xylan (Sigma) L^-1^ was suspended in 50 mM acetate buffer (pH 5.5). The resultant mixture was then incubated at 50°C for 30 min followed by quickly chilling on ice. The amount of reducing sugars released was measured by the 3,5-dinitrosalicylic acid (DNS) method [38]. One unit of xylanase activity was defined as the amount of xylanase required to release 1 μmol D-xylose per min under the assay conditions.

### Screening of D-xylose-fermenting yeasts

Durham tubes test was used to test the ability of a strain, which was isolated from the guts of *R*. *chinensis* to ferment D-xylose. The tubes containing YPX medium (1% yeast extract, 2% peptone, 2% D-xylose) were inoculated with a cell optical density (OD_600_) adjusted at 0.05. The inoculated tubes were incubated at 25°C on an orbital shaker at 125 rpm up to 28 days and observed daily for the qualitative production of gas [39]. D-xylose fermentation was considered positive when the production of gas was detected.

### Analytical methods

Strains showed positive results in Durham tubes were then subjected to fermentation assays in the YPX culture medium as described previously [27,40]. Samples were taken at 0, 12, 24, 48 and 72 h. Cell concentration was determined by correlating optical density measurements taken with a UV-visible spectrophotometer (Shimadzu-UV2600, Japan) at 600 nm with a constructed calibration curve (dry weight x optical density). The cells were recovered by centrifugation at 2600 xg for 15 min. The same steps were repeated in tubes containing YP medium (same components of YPX without D-xylose) as a negative control in order to verify the possibility of alcohol production from yeast extract and peptone. The supernatant obtained was analyzed for ethanol by potassium dichromatic method [41] using different concentrations (1–10% v/v) of absolute ethanol as standard, and sugar consumption by 3,5-dinitrosalicylic acid (DNS) method [38]. Ethanol yield (g/g) was defined as the ratio between ethanol concentration (g/L) and sugar consumed (g/L). Ethanol productivity was calculated as the ratio of maximum ethanol concentration (g/L) to the respective fermentation time (h). The maximal theoretical yield of ethanol was defined as 0.51 g ethanol per g xylose (1.67 mol of ethanol per mol of xylose). The efficiency of sugar conversion to ethanol (%) was calculated as the ratio between ethanol yield (g/g) and the maximal theoretical yield of ethanol. Sugar consumption (%) was determined as a percentage of the initial sugar concentration [42].

### Statistical analysis

Results are presented as mean ± standard deviation (SD) of three replicates. The statistical analyses were carried out using SPSS-20. Data obtained were analyzed statistically to determine the degree of significance using one-way analysis of variance (ANOVA) and t-test at a probability level (*P*) ≤ 0.05.

## Results and discussion

### Isolation, identification and diversity of yeasts

On the basis of phenotypic characteristics and sequence analyses of the D1/D2 domains and ITS regions, 18 yeast species were successfully identified. Table 1 demonstrated a list of those yeast species isolated from termite guts, where it also showed the results for qualitative and quantitative assays of xylanase production as the relevant observations for D-xylose fermentation inside Durham tubes. A total of 92 yeast strains were actually isolated and identified from termite gut samples. Of those, 40 and 32 strains were obtained following growth in YNB-D-xylose and YNB-xylan media, respectively. Out of 18 yeast species, 11 were previously known and seven were novel (*C*. *gotoi*, *C*. *pseudorhagii*, *Hamamotoa lignophila*, *Meyerozyma guilliermondii*, *Sugiyamaella* sp. 1, *Sugiyamaella* sp. 2, *Sugiyamaella* sp. 3). As shown in Table 1, all yeasts belonged to Ascomycota, except *Sterigmatomyces halophilus*, *H*. *lignophila* and *Vanrija humicola* that were actually belonged to Basidiomycota.

**Table 1:** Identification of xylanase-producing and/or D-xylose-fermenting yeasts isolated from wood-feeding termite, *R*. *chinensis*.

Yeast species	Sampled medium		D-xylose fermentation in Durham tube	Xylanase production	Xylanase activity (U)^C^	
	YNB-D-xylose (n = 30)	YNB-xylan (n = 30)			YNB-D-xylose	YNB-xylan
***Barnettozyma californica***	0	1	-	+(1) ^B^	0.00±0.00^a^	0.32±0.03^a^
***Candida* sp.**	1	3	-	+(3)	0.07±0.01^b^	0.98±0.08^b^
***Candida gotoi***^A^	3	1	-	+(1)	0.10±0.02^c^	1.13±0.03^c^
***Candida pseudorhagii***^A^	6	4	+ (3)	+(3)	0.98±0.08^c^	1.73±0.09^d^
***Candida silvanorum***	0	1	-	-	0.00±0.00^a^	0.00±0.00^e^
***Candida tropicalis***	5	3	-	-	0.00±0.00^a^	0.00±0.00^e^
***Cyberlindnera bimundalis***	1	0	-	-	0.00±0.00^a^	0.00±0.00^e^
***Cyberlindnera* sp.**	0	3	-	+(2)	0.00±0.00^a^	0.90±0.20^b^
***Debaryomyces hansenii***	2	0	-	-	0.23±0.03^d^	0.00±0.00^e^
***Hamamotoa lignophila***^A^	5	4	+ (5)	+(2)	0.35±0.01^e^	0.68±0.04^f^
***Meyerozyma guilliermondii***^A^	2	3	+ (2)	+(1)	0.20±0.01^f^	0.78±0.02^g^
***Sterigmatomyces halophilus***	2	2	-	+(2)	0.11±0.01^c^	0.31±0.02^a^
***Sugiyamaella smithiae***	6	1	-	+(3)	0.90±0.03^c^	0.93±0.05^b^
***Sugiyamaella* sp.1**^A^	4	2	+ (2)	+(4)	0.89±0.02^c^	1.01±0.12^b^
***Sugiyamaella* sp.2**^A^	2	1	-	-	0.00±0.00^a^	0.00±0.00^e^
***Sugiyamaella* sp.3**^A^	0	2	-	+(1)	0.00±0.00^a^	0.70±0.03^g^
***Vanrija humicola***	0	1	-	+(1)	0.00±0.00^a^	0.72±0.07^fg^
***Wickerhamomyces* sp.**	2	0	-	+(1)	0.43±0.03^g^	0.00±0.00^e^

^**A)**^ Novel yeast species isolated from *R*. *chinensis*.

^**B)**^ Number in parenthesis represent the number of yeast strains positive for ethanol production from D-xylose fermenting yeasts and/or xylanase production.

^**C)**^ Xylanase activities (U/mL) were obtained from cell-free supernatants induced by a carbon source (D-xylose or xylan) for 3 days at 30°C.

Means with the same letters in the same column showed insignificant difference (*P* ≤ 0.05).

Values are the mean of three replicates ± SD.

The yeasts from genus *Candida* were the most frequently occurring ones, represented by five different species (*Candida* sp., *C*. *gotoi*, *C*. *pseudorhagii*, *C*. *silvanorum*, *C*. *tropicalis*), followed by four *Sugiyamaella* yeast species (*S*. *smithiae*, *Sugiyamaella* sp. 1, *Sugiyamaella* sp. 2, *Sugiyamaella* sp. 3), and two yeast species of *Cyberlindnera* (C. *bimundalis*, *Cyberlindnera* sp.). The yeast species, *C*. *pseudorhagii* (*Hyphopichia* clade), was the most frequently isolated species from termite gut samples, representing six and four samples successfully cultured on YNB-D-xylose and YNB-xylan media, respectively. The yeast species, *H*. *lignophila* (*Microbotryum* clade), was the second most frequently isolated one, occurring in five gut samples cultured on YNB-D-xylose and four samples on YNB-xylan medium, followed by the yeasts of *C*. *tropicalis* (*Lodderomyces/Spathaspora* clade), and *S*. *smithiae* (*Sugiyamaella* clade) observed in five and six samples on YNB-D-xylose medium as well as three and one samples cultured on YNB-xylan medium, respectively.

Yeast isolations from the gut of insects have led to discovering a large number of new species of yeasts. Regardless of these previous efforts, the confirmation of various symbiotic yeasts from insect guts and their novel functions remains largely understudied. Wood-feeding termite guts represent a unique and excellent model due to their highly structured microenvironments. The efforts made by Starmer and his colleagues for more than 30 years have revealed a widespread association between yeasts and their host insects in various habitats. As a matter of fact, *Barnettozyma californica*, *Wickerhamomyces* sp. and *Debaryomyces hansenii*, were primarily isolated from the source of rotten wood [43]. The yeast species, *C*. *pseudorhagii* and *M*. *guilliermondii*, were indeed associated with the insect frass [44,45]. Some salt-tolerant yeast (*S*. *halophilus*) species were commonly isolated from the marine environments [46]. However, the yeast of *S*. *halophilus* strain SSA-1575^T^ (KX791366), a type of xylanase-producing yeast strains, was successfully isolated from a wood-feeding termite guts, *R*. *chinensis*. Clearly, it can be speculated that a unique function of the yeasts may largely depend on their living environments that may present an evolutionarily imposed pressure on their yeast function development.

### Xylanase-producing and D-xylose fermenting-yeasts

Twenty-five strains from 13 yeast species listed in Table 1 were identified to be a xylanase producer on xylan-agar medium, which were mainly represented by the known yeast species, such as *B*. *californica*, *Candida* sp., *Cyberlindnera* sp., *S*. *halophilus*, *S*. *smithiae*, *V*. *humicola* and *Wickerhamomyces* sp. However, some novel yeast species isolated from wood-feeding termite guts, *R*. *chinensis* were also firstly to be confirmed with this important property, such as *C*. *gotoi*, *C*. *pseudorhagii*, *H*. *lignophila* and *M*. *guilliermondii*, *Sugiyamaella* sp. 1, and *Sugiyamaella* sp. 3. The well-known *C*. *tropicalis* and *M*. *guilliermondii* isolates obtained from a piece of the decayed wood were not to be confirmed as a xylanase producer in the plate assays as reported in early reference [43], although both of them have been thoroughly studied regarding their capability in the utilization of pentose sugars (D-xylose and L-arabinose) [47]. In the present study, the members of *Sugiyamaella* genus isolated from termite *R*. *chinensis* were identified for the first time as a xylanase producer. Recently, the novel *Sugiyamaella* species, *S*. *xylanolytica* [43], and *S*. *smithiae* isolated from a piece of the decaying wood were further reported with a xylanolytic activity [24]. Indeed, the yeast strains of *C*. *gotoi*, *C*. *pseudorhagii*, and *H*. *lignophila* listed in Table 1 have been shown with an important property to produce xylanases. Interestingly, no previous reports were documented with this characteristic for the yeast species as mentioned above.

Out of 18 yeast species isolated from termite *R*. *chinensis*, four novel yeast species, *C*. *pseudorhagii*, *H*. *lignophila*, *M*. *guilliermondii*, and *Sugiyamaella* sp. 1 in Table 1 showed gas formation (CO_2_) in Durham tubes that is an indicator of D-xylose fermentation. Currently, there is no other report that has been documented with this property for yeast species of *C*. *pseudorhagii* or *H*. *lignophila* in fermenting D-xylose until the present study. However, there are a few reports, beside our observations, documented for two new *Sugiyamaella* species (*S*. *xylanicola* and *Sugiyamaella* sp. 1) isolated from a piece of the decayed wood that was confirmed with a capability to ferment D-xylose into ethanol [43]. D-xylose fermentation property has already been reported for the yeast of *M*. *guilliermondii* [31,34]. Interestingly, no gas formation was observed for the yeast species, *C*. *tropicalis*, when D-xylose was served as the sugar substrate in Durham tubes although this species has previously been described to ferment D-xylose, producing gas from pentose sugar [48].

### Ethanol production from D-xylose fermentation

To evaluate the ethanol production from D-xylose fermentation, four strains of the xylanase-producing yeast species were selected to commit a fermentation assay in D-xylose-supplemented YPX culture medium (50 g/L). The fermentation parameters (ethanol concentration, D-xylose consumption, ethanol yield, ethanol productivity, fermentation efficiency) are summarized in Table 2. Fermentation performance revealed that all strains tested were able to consume D-xylose, with the consumption rates ranged from 77.6% to practically 95.2% in 48–72 h. As a matter of fact, the ethanol production was observed during the yeast fermentation assay, where the highest ethanol concentration was recorded at 14.7 g/L in 48 h by the *Candida pseudorhagii* sp. nov. strain SSA-1542^T^, followed by *Hamamotoa lignophila* sp. nov. strain SSA-1576^T^ at 10.1 g/L in 72 h. However, *Sugiyamaella* sp.1 nov. strain SSA-1592^T^ and *Meyerozyma guilliermondii* sp. nov. strain SSA-1522^T^ showed a low ethanol concentration at 4.6 g/L or 3.8 g/L, respectively. The variations in ethanol production among these yeast strains tested may probably be associated with their own physiological differences. Compared with the referenced yeasts in Table 2, *C*. *pseudorhagii* sp. nov. strain SSA-1542^T^ showed a competitive capability in fermenting D-xylose substrate, where it presented the highest ethanol concentration in a shorter period of fermenting time (48 h). In addition, this yeast strain was also characterized by its high ethanol yield (0.31g/g), high ethanol productivity (0.31 g/L·h), as well as a relatively high fermentation efficiency (60.7%).

**Table 2:** The comparison of D-xylose fermentation performance for ethanol production between standard yeasts and the yeasts from termite gut system of *R*. *chinensis*.

Yeast strain	Ethanol concentration (gL^-1^)	Xylose consumption* (%)^a^	Ethanol yield (gg^-1^)^b^	Fermentation efficiency (%)^c^	Ethanol productivity (gL^-1^h^-1^)^d^	Fermentation time (h)^e^	Reference
***Candida blankii* ATCC 18735**^**T**^	5.1	45.5	0.10	19.6	0.07	72	[49]
***Candida shehatae* NRRL Y-12856**^**T**^	24.0	100.0	0.48	94.1	0.29	82	[50]
***Pichia stipitis* CBS 5776**^**T**^	22.3	100.0	0.45	88.2	0.34	65	[51]
***Schizosaccharomyces pombe* ATCC 2478**^**T**^	5.0	34.3	0.10	19.6	0.07	72	[49]
***Candida pseudorhagii* sp. nov. strain SSA-1542**^**T**^ **(KX907628)**^g^	14.7	95.2	0.31	60.7	0.31	48	This study
***Hamamotoa lignophila* sp. nov. strain SSA-1576**^**T**^ **(KU513951)**^h^	10.1	90.6	0.22	43.1	0.14	72	This study
***Meyerozyma guilliermondii* sp. nov. strain SSA-1522**^**T**^ **(KX791408)**^m^	3.8	77.6	0.10	19.6	0.08	48	This study
***Sugiyamaella* sp.1 nov. strain SSA- 1592**^**T**^ **(KX791379)**^n^	4.6	81.3	0.11	21.6	0.06	72	This study

*Initial xylose concentration (gL^-1^) is 50.

^a^ Xlose consumption (%): percentage of initial xylose consumed.

^b^ Ethanol yield (gg^-1^): ratio between ethanol concentration (gL^-1^) and xylose consumed (gL^-1^).

^c^ Fermentation efficiency (%): percentage of the maximal theoretical yield of ethanol (0.51 g ethanol/g xylose).

^d^ Ethanol productivity (gL^-1^h^-1^): ratio of ethanol concentration (gL^-1^) and fermentation time (h).

^e^ Fermentation time (h): a time when the maximum ethanol production (gL^-1^) value was attained.

^g,^ Cell concentration (gL^-1^) is 9.0.

^h^ Cell concentration (gL^-1^) is 9.8.

^m^ Cell concentration (gL^-1^) is 7.5.

^n^ Cell concentration (gL^-1^) is 8.4.

### Xylanolytic yeasts

A high xylanolytic activity under xylan substrate induction was observed for some yeast strains isolated from termite guts. The yeast of *C*. *pseudorhagii* showed the highest xylanolytic activity at 1.73 U/mL and 0.98 U/mL with xylan and D-xylose, respectively (Table 1), which were followed by *C*. *gotoi*, *Sugiyamaella* sp. 1, *Candida* sp., *S*. *smithiae*, *Cyberlindnera* sp., *M*. *guilliermondii* and *H*. *lignophila*. However, no any xylanolytic activity was actually detected for the yeast of *Wickerhamomyces* sp. under a xylan induction (D-xylose, 0.43 U/mL). As a matter of fact, all yeast species tested demonstrated a relatively low xylanase activity when using D-xylose as an inducer (Table 1). When using the YNB-xylan as a substrate inducer, the yeast of *S*. *smithiae* SSA-1590^T^ (KX791377) from termite guts presented a higher xylanase activity (0.93 U/mL) than that of *S*. *smithiae* UFMG-HM-80.1 (0.50 U/mL) from decaying wood [24] and *S*. *xylanicola* sp. nov. (0.15 U/mL) from rotten-wood samples [26]. It is noteworthy that the xylanolytic activity of many yeast species from termite guts have been shown a better performance with a xylan induction substrate than that of D-xylose [24,26] although xylanases can be usually induced by various inducing substrates, such as xylan, xylobiose, xylose, as well as xylooligosaccharides [52]. It has also been reported that only less than 25 yeast species out of ~1500 described yeasts were confirmed with an ability to ferment D-xylose and produce ethanol, and further, the xylanases production is really restricted to a few unique yeast species [53].

### Description of *Candida pseudorhagii* Ali, Wu & Sun sp. nov.

*C*. *pseudorhagii* Ali, Wu & Sun sp. nov. (urn:lsid:imycobank.org:names: MycoBank accession number MB 821228).

#### Phylogeny of *C*. *pseudorhagii* sp. nov. strain SSA-1542^T^

As a novel yeast species isolated from termite gut, *C*. *pseudorhagii* has exhibited some unique and impressive properties that are potentially valuable for biorefinery purpose, such as the highest xylose fermentation capability as well as its associated xylanase activity among 18 yeast species identified from termite guts. Based on these reasons, the yeast strain, designated as SSA-1542^T^ from *C*. *pseudorhagii*, was actually selected for a further discussion regarding its phylogeny and physiology.

Pairwise sequence analysis revealed that three yeast strains from *C*. *pseudorhagii* sp. nov., SSA-1542, SSA-1542S, and SSA-1542SS with a xylanase-producing property, were genetically conspecific (100% identity in both D1/D2 domains and ITS region sequencing). With a further analysis, a novel yeast species can be indeed confirmed in terms of their morphology, physiology, and blast analysis; and the closest sequences in the available database of GeneBank propose them relevant to those yeasts from *C*. *pseudorhagi* isolate NRRL YB-2076^T^ (AY789656) with 88% identity in the large-subunit rDNA (57 nucleotide substitutions and 3 gaps), and *H*. *heimii* strain WM 07.74^T^ (FM178347) with 97% identity in the ITS region (6 substitutions and 3 gaps). Based on these analyses, the type strain of *C*. *pseudorhagii* sp. nov. SSA-1542^T^ can be confirmed as a novel yeast species with their accession numbers KX907628 and KX791416 using the D1/D2 domain and ITS region sequences, respectively.

The phylogenetic placement of this novel species based on D1/D2 sequences was shown in Fig 1. BLAST search showed that SSA-1542^T^ significantly differed from its closest relatives, a undescribed species, *Hyphopichia* sp. strain NYNU 14666^T^, by 14% sequence divergence (68 substitutions and 6 gaps) and one from *C*. *gotoi* strain CBS 8531 by 14.8% sequence divergence (80 substitutions and 3 gaps) in the same cluster. Although the position of this novel species within the *Hyphopichia* clade remained unclear due to the low bootstrap value, the same tree topology was derived from the maximum-parsimony and minimum-evolution analyses (data not shown). Furthermore, D1/D2 sequences from its three closest known species within the *Hyphopichia* clade (*Pichia heimii* strain D123, *Hyphopichia heimii* strain NRRL Y-7502^T^ and *Candida rhagii* strain D43_pir) exhibited 11.9–14.1% sequence divergence (60–71 substitutions and 4–7 gaps). Such degree of variations is considered sufficient to distinguish the stain SSA-1542^T^ as a separate novel species distincted from presently described and undescribed species [54].

In addition to molecular differences, SSA-1542^T^ differed from the currently recognized species in the *Hyphopichia* clade in terms of its physiological characteristics presented in Table 3. Our data prompted us to compare our representative yeast strain with those closed relevant reference yeasts in the same clade. *H*. *heimii* was introduced by Kurtzman [44] as a new combination in the genus *Hyphopichia* and proposed a novel species namely, *C*. *pseudorhagii*, in terms of its genotypic and phenotypic characteristics. At present, it has been confirmed that the six *Candida* species (*C*. *gotoi*, *C*. *rhagii*, *C*. *pseudorhagii*, *C*. *fennica*, *C*. *homilentoma* and *C*. *khmerensis*) and also three species of *Hyphopichia* (*H*. *heimii*, *H*. *burtonii* and *H*. *pseudoburtonii*) belong to the clade of *Hyphopichia* [19,44].

**Table 3:** Comparison of physiological traits of *C*. *pseudorhagii* sp. nov. strain SSA-1542^T^ with three closely related species in *Hyphopichia* clade.

Traits	1	2^a^	3^b^	4^c^	Traits	1	2^a^	3^b^	4^c^	Traits	1	2^a^	3^b^	4^c^
**Fermentation**														
D-galactose	+	+	+/-	+	Cellobiose	+/-	n	n	n	Starch	-	n	n	n
Maltose	-	+/-	-	-	Melezitose	-	n	n	n	D-xylose	+	n	n	n
α, α-trehalose	+	+	+	-	Raffinose	+	-	+/-	-					
Melibiose	-	n	n	n	Inulin	-	n	n	n					
**Carbon assimilation**														
L-sorbose	-	-	+/-	-	α-methyl-D-glucoside	+	+	+/-	+	L-arabinitol	w, d	n	n	n
D-glucosamine	+	+	+	-	Arbutin	-	n	n	n	Galactitol	-	+	-	+
D-ribose	+	+	+/-	+	Soluble starch	-	-	-	+	D-gluconate	+	+/-	+/-	-
L-arabinose	+	+	+/-	+	Glycerol	+	+	+	+	DL-lactate	-	-	+/-	+
D-arabinose	+/-	-	-	-	Erythritol	+	+	+/-	+	Citrate	-	+	-	+
L-rhamnose	+	+	+/-	+	Xylitol	+/-	n	+	+					
**Nitrogen assimilation**														
Nitrate (potassium)	+	-	-	-	L-lysine	-	n	+	+	Creatinine	+	n	n	n
Nitrite (sodium)	-	n	-	-	Cadaverine	+	n	+	+	Imidazole	+	n	n	n
Ethylamine	-	n	+	+	Creatine	+	n	n	n	D-tryptophan	d	n	n	n
**Vitamin requirements**														
Vitamin-free	+	+	+/-	+										
**Growth tests**														
35°C	+	n	n	+	Acetic acid (1%)	+	n	n	n	NaCl (16%)	+	n	n	n
37°C	+	-	-	+	D-glucose (50%)	+	n	+/-	+					
Cycloheximide (0.01%)	-	n	-	-	D-glucose (60%)	+	n	n	n					
Cycloheximide (0.1%)	-	n	-	-	NaCl (10%)	+	+	n	+					
**Other tests**														
Acetic acid production	+	n	n	n	Urea hydrolysis	+/-	n	n	n					

**1**: *C*. *pseudorhagii* sp. nov. strain SSA-1542^T^;

**2**: *Hyphopichia heimii*;

**3**: *Candida rhagii*;

**4**: *Candida gotoi*.

The following traits are invariable in the *C*. *pseudorhagii* sp. nov. and closely related species described: fermentation of glucose (+), sucrose (+), lactose (-); assimilation of D-glucose (+), D-galactose (+), D-xylose (+), sucrose (+), maltose (+), trehalose (+), cellobiose (+), salicin (+), melibiose (-), lactose (-), raffinose (+), melezitose (+), inulin (-), ribitol (+), D-glucitol (+), D-mannitol (+), *myo*-inositol (-), N-acetyl-D-glucosamine (+), 2-keto-D-gluconate (+), succinate (+), methanol (-), ethanol (+), hexadecate (+); growth at 25°C and 30°C (+). Starch-like substances were not formed. Diazonium blue B (DBB) reaction is negative. Gelatin liquification is negative.

^a^ Data from the CBS Yeast Database (http://www.cbs.knaw.nl/yeast/BioloMICS.aspx).

^b^ Data from the CBS Yeast Database (http://www.cbs.knaw.nl/yeast/BioloMICS.aspx).

^c^ Data from the CBS Yeast Database (http://www.cbs.knaw.nl/yeast/BioloMICS.aspx).

+, positive; -, negative, +/-, variable; w, weakly positive; d, delayed positive; n, data not available or not tested.

#### Morphological characterization of *C*. *pseudorhagii* sp. nov. strain SSA-1542^T^

**Fig 2:**
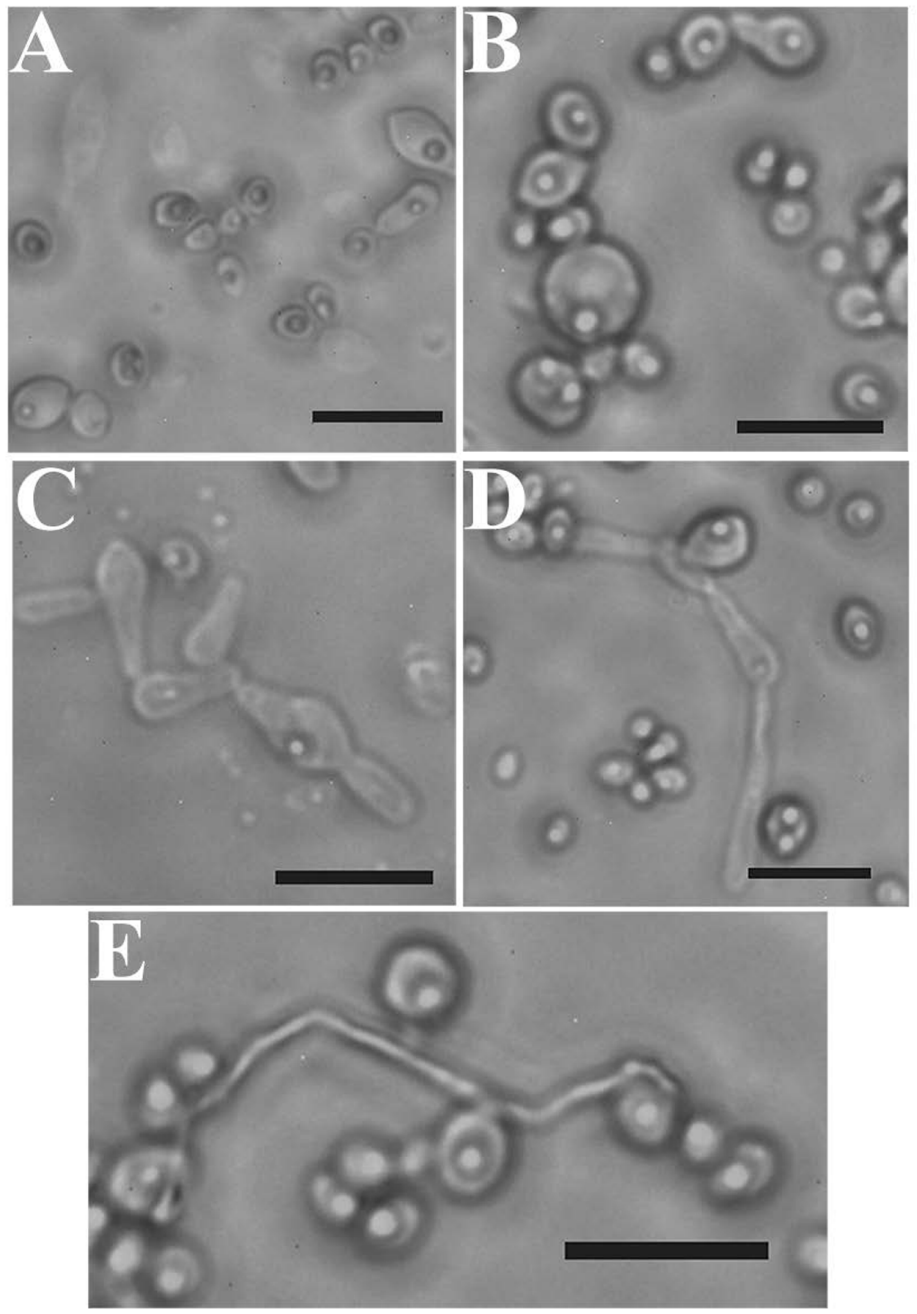
Morphological characterization of *C*. *pseudorhagii* sp. nov. strain SSA-1542^T^. Budding yeast cells after 3 days on YM broth (**A**) and after 7 days on YM agar (**B**), at 25°C. Pseudohyphal formation on a Dalmau plate culture under the coverglass cells, after 10 days on cornmeal agar at 25°C (**C, D**). True hyphae bearing blastoconidia after 30 days on corn meal agar at 25°C (**E**). Bar 10 μm.

In order to complete the morphological identification of the novel yeast strain, *C*. *pseudorhagii* SSA-1542^T^ was selected for further observation and characterization (Fig 2). The cells of this strain are mostly spherical to elongate (1.5–5.6 x 1.8–6.5 μm) in YM broth after 3 days at 25°C; occurring singly, in pairs, or in clusters; asexual reproduction by multilateral budding (Fig 2A and 2B) and sparsely pseudohyphae are present. Colonies are white to creamy color, butyrous, semi-glistening and have a clear margin on YM agar after 7 days at 25°C. After 10 days of yeast growth on cornmeal agar in Dalmau plates, sparsely to abundantly pseudohyphae bearing blastoconidia were present (Fig 2C and 2D). Additionally, some tapered denticulate outgrowths which bear blastoconidia on the denticles were detected. True hyphae were not observed on YM agar or in Dalmau plates, but well developed true hyphal outgrowth bearing blastoconidia was observed after 30 days on cornmeal agar in Dalmau plates (Fig 2E). The cultures produce a faint ester-like odor. Ascospores and conjugations not observed. *Candia pseudorhagii* SSA-1542^T^ is designated as the type strain, isolated from the gut of *Reticulitermes chinensis* Synder (Isoptera: Rhinotermitidae) found on rotting wood trees at Huazhong Agricultural University, Wuhan, China. The species etymology name *pseudorhagii* denotes that this species is in a close relationship with *Candida rhagii*.

### The comparison of ethanol and xylanase productivity between the yeast strain SSA-1542^T^ and those of engineered yeast strains

Tables 4 and 5 were constructed to show the superiority of the wild-type novel strain, *C*. *pseudorhagii* SSA-1542^T^, in its productivity of xylanase and ethanol compared with those of recombinant strains published in other studies from references. The efficient conversion of xylan to ethanol can be accomplished by degrading xylan to xylose. Although *S*. *cerevisiae* is the most widely used species for the heterologous production of xylanase and xylosidase enzymes, a major disadvantage associated with this yeast species, particularly for its wild-type, is its inability to utilize xylose. Therefore, wild-type strains of *S*. *cerevisiae* have been genetically engineered to assimilate xylose [55]. While recombination changes have facilitated the ability of this yeast for a direct fermentation of xylan [56], the conversion rate of xylan into xylose is considered to insufficient and the alternative ways are indeed required in order to increase hemicellulose degradation activity [55]. In this regard, Sakamoto et al. [55] successfully constructed a xylose-assimilating *S*. *cerevisiae* capable of co-displaying endoxylanase, β-xylosidase and β-glucosidase from *Trichoderma reesei*, *Aspergillus oryzae* and *Aspergillus aculeatus*, respectively. They claimed that ethanol production with the yeast of *S*. *cerevisiae* can be significantly improved by a genetic modification processing. However, some wild-type of the yeasts explored from some unique niches in nature, particularly from the guts of wood-feeding termites, presented a super performance in xylose fermentation and ethanol conversion capability prior to having any genetic modification.

**Table 4:** Ethanol yield and ethanol productivity in the wild-type strain SSA-1542^T^ compared to those of the recombinant *S*. *cerevisiae* strains.

Yeast strain	Relevant features	Ethanol yield (gg^-1^)	Ethanol productivity (gL^-1^h^-1^)	Reference
**SSA-1542**^**T**^	Wild-type *C*. *pseudorhagii* species novel strain	0.31	0.31	This study
**Y22-3**	Engineered *S*. *cerevisiae* strain for anaerobic fermentation of xylan	NA	0.002	[57]
**Y127**	Engineered *S*. *cerevisiae* strain for anaerobic fermentation of xylan	NA	0.009	[57]
**Y128**	Engineered *S*. *cerevisiae* strain for anaerobic fermentation of xylan	NA	0.167	[57]
**MT8-1/pUCSXIIXA/pWX1X2XK**	Codisplay of XYNII and XylA in xylose-assimilating yeast	0.29	NA	[56]
**1400(pLNH30)**	Genetically engineered *S*. *cerevisiae* to conferment xylose	0.29	NA	[58]
**TMB3250**	Recombinant xylose-fermenting *S*. *cerevisiae*	0.30	NA	[59]
**NBRC1440X**	Mating recombinant xylose-fermenting *S*. *cerevisiae* strain	NA	0.32	[60]
**CCUGS3310-X**	Metabolic engineered *S*. *cerevisiae* strain	0.09	0.07	[22]
**YRH403**	Industrial *S*. *cerevisiae* strain engineered to ferment xylose	0.18	0.032	[61]
**YRH392**	Industrial *S*. *cerevisiae* strain engineered to ferment xylose	0.22	0.038	[61]
**YRH394**	Industrial *S*. *cerevisiae* strain engineered to ferment xylose	0.22	0.035	[61]
**YRH388**	Industrial *S*. *cerevisiae* strain engineered to ferment xylose	0.23	0.058	[61]
**YRH400**	Industrial *S*. *cerevisiae* strain engineered to ferment xylose	0.24	0.075	[61]
**YRH390**	Industrial *S*. *cerevisiae* strain engineered to ferment xylose	0.25	0.068	[61]
**YRH396**	Industrial *S*. *cerevisiae* strain engineered to ferment xylose	0.27	0.081	[61]
**MT8-1/Xyl**	Recombinant xylose-assimilating yeast strain	0.37	0.06*	[63]
**MT8-1/Xyl/BGL**	Recombinant xylose-assimilating yeast strain	0.37	0.05*	[63]
**TMB3001**	Recombinant xylose-fermenting *S*. *cerevisiae*	0.30	NA	[59]
**FPL-YSX3**	Recombinant xylose-fermenting *S*. *cerevisiae* strain	0.12	NA	[65]
**FPL-YS10**	Recombinant xylose-fermenting *S*. *cerevisiae* strain	0.00	0.00	[65]
**FPL-YS1020**	Recombinant xylose-fermenting *S*. *cerevisiae* strain	0.00	0.00	[65]

NA, Not Available.

*The specific ethanol production rate unit is gram of ethanol per gram of dry cell weight per hour.

**Table 5:** Xylanase activity of the wild-type novel strain SSA-1542^T^ compared to that of recombinant strains.

Yeast strain	Relevant features	Xylanase activity (UmL^-1^)	Reference
**SSA-1542**^**T**^	Wild-type *C*. *pseudorhagii* species novel strain	1.73	This study
**pCE3**	Recombinant *S*. *cerevisiae* expressing *Aureobasidium pullulans xynA*	1.68	[67]
**pGE3**	Recombinant *S*. *cerevisiae* expressing *A*. *pullulans xynA*	1.60	[67]
**Cfxyn1p**	Recombinant xylanase CfXYNl gene expressed in *S*. *cerevisiae*	1.70	[68]
**Xyn10A**	Recombinant Xyn10A expressed in *Flavobacterium johnsoniae*	0.83	[69]
**Xyn10AΔFn3**	Recombinant Xyn10A expressed in *F*. *johnsoniae*	0.31	[70]
**HB101(pBR322)**	Recombinant *Thermomonospora fusca* gene in *E*. *coli*	< 0.01	[70]
**HB101(pGG92)**	Recombinant *T*. *fusca* gene in *E*. *coli*	< 0.01	[70]
**TK24(pGG82)**	Recombinant *T*. *fusca* gene in *E*. *coli*	< 0.10	[70]
**HB101(pGG93)**	Recombinant *T*. *fusca* gene in *E*. *coli*	0.15	[70]
**HB101(pTX101)**	Recombinant *T*. *fusca* gene in *E*. *coli*	0.27	[70]
**MT8-1/pCAS1-XYNII**	Recombinant cell-surface xylanase in *S*. *cerevisiae*	0.00	[66]
**MT8-1/pCAS1-RGSHis6-XYNII**	Recombinant cell-surface xylanase in *S*. *cerevisiae*	0.00	[66]

The higher concentration of catabolized xylose along with its faster consumption rate (Table 2) may suggest two possible biochemical mechanisms in the superiority of the novel wild-type yeast strain SSA-1542^T^ in this study. One possibility of the increased xylose consumption is due to the efficient xylose transport, without further metabolic conversion of xylose to other intermediates [57]. The other possibility suggests that SSA-1542^T^ has pentose phosphate enzyme or more active xylose catabolism, or both, possibly allowing an enhanced conversion of xylose to ethanol [57]. Clearly, with both possible unique mechanisms occurred in the novel strain of SSA-1542^T^, the ethanol productivity can be enhanced significantly. As shown in Table 4, the ethanol production performed by the engineered *S*. *cerevisiae* Y128, Y22-3, and Y127 strains from references was much lower than that of the wild-type yeast strain SSA-1542^T^. As another evidence from Table 4, the ethanol yield produced by SSA-1542^T^ was also relatively higher than that of the recombinant yeast strains MT8-1/pUCSXIIXA/pWX1X2XK, 1400(pLNH30), and TMB3250 [56,58,59]. Although the ethanol productivity of the wild-type strain SSA-1542^T^ was much similar to the recombinant xylose-fermenting strain NBRC1440X from reference [60], we still have an opportunity to improve its fermentation performance with a genetic modification to meet a higher requirement from the biorefinery industry.

In addition, the mechanism of xylitol accumulation is another evidence that may support the superiority of the wild-type strain SSA-1542^T^ in its ethanol productivity. As shown in Table 4, the engineered *S*. *cerevisiae* strains; such as CCUGS3310-X, YRH388, YRH390, YRH392, YRH396, YRH400, YRH403, MT8-1/Xyl/BGL and MT8-1/Xyl showed less ethanol productivity. This result clearly confirmed that much of the xylose was converted to the xylitol (side-product) by these engineered yeast strains, which lowered the final yields of ethanol when compared with that of the wild-type strain SSA-1542^T^ [22,61–63]. The high xylose reductase (XR)/ xylitol dehydrogenase (XDH) activity ratios of the engineered yeast strains may contribute to a very high xylitol accumulation [64]. In a previous study of the ethanol production from xylose by a recombinant *S*. *cerevisiae*, it was confirmed that an appropriately low-level expression of D-xylulokinase gene *XYL3* increased ethanol productivity and xylose uptake but decreased xylitol accumulation as a by-product during xylose fermentation process [65].

In general, the higher xylanolytic activity could be attributed to the action of a microorganism able to decompose polysaccharides such as xylan efficiently [26]. Xylan is the main component of hemicellulose, that mainly consists of xylose. Although xylanases are usually induced by xylan, D-xylose, xylobiose or xylooligosaccharides [66], xylanolytic activities in yeasts have been shown to be better induced by xylan substrate as a source of carbon than by D-xylose, which were evidenced by both in the present study and other reports with particular wild-type yeast strains [24,26]. As an indicator of xylanolytic activity, the xylanase activity of the wild-type SSA-1542^T^ strain from our study was higher than that of the recombinant *S*. *cerevisiae* strains published in other studies [67–69] (Table 5). The lack of xylanolytic activity in five transformed strains grown on xylan could indicate that the activity binds to undegraded xylan [70]. As a matter of fact, the xylanolytic activity performed by the strain SSA-1542^T^ can be potentially enhanced further because current activity was actually observed under the conditions that had not been optimized in terms of its culture conditions, such as pH, temperature, etc.

Clearly, as shown in the constructed Tables 4 and 5, the wild-type SSA-1542^T^ strain had a remarkable performance in its productivity of xylanase and ethanol when compared with those recombinant strains from references. This novel strain could be a new source of genes to be used for constructing a better engineering strain for other yeast species, such as *S*. *cerevisiae*. which may also serve as a promising yeast candidates applied for the advancement of biorefinery industry.

### Yeast symbionts and the association with their hosts

The diversity of gut-inhabiting bacteria from certain wood-feeding insects, such as termites, is well known, and their phenotypic and genotypic characterizations of the symbiotic relationships have shown a close connection between hosts and their symbionts. However, the associations between fungi and insects are less well known [71]. It is not clear if the insect-associated yeasts were transient, acquired from a feeding substrate, or presented as an obligated symbiotic relationship. The wood-feeding termite, *R*. *chinensis*, is a widely distributed wood-feeding termite species in China, which damages the wooden materials, such as dry timber and vegetable fiber, by building termite nests in soil and xylem of living old trees [54]. The relatedness between rotten wood and wood-feeding termites raises an interesting question regarding the role of those symbiotic yeasts in the gut of termites. Are the yeasts performing some essential roles in the gut or are they merely acquired during digestion of yeast-infected wood? As a matter of fact, *C*. *pseudorhagii* strain SSA-1542^T^ sp. nov., with its closest members of *Hyphopichia* clade (Table 3), the majority of strains isolated from *R*. *chinensis* in this study (unpublished data), are commonly able to assimilate D-xylose, cellobiose, and salicin [72]. These data suggest that the indicator substrates, such as xylose, associated with a variety of enzymes may truly involve in a metabolic pathway in the digestion processes on woody substrates, as well as the following fermentation steps occurred in the termite gut system [73]. The microbial decomposition of cellulose provides many intermediate products such as cellobiose and glucose that can be directly used by the host insects or further used as a substrate for fermentation by yeasts [71]. The xylanases involved in the decomposition of hemicellulose in termite digestive system may partially generate from the symbiotic yeasts in the host termites [74]. Interestingly, the identified 13 yeast species listed in Table 1 were confirmed as a xylanase producer, where five novel yeast species were firstly to be verified with their property for xylanase production. In general, hemicellulose substrates include a variety of heteropolysaccharides such as, xylans, mannans, glucans, arabinans and galactans. But, xylan is usually a dominant sugar presented in hemicelluloses of the soft woods commonly used by wood-feeding termites [4]. The microbial decomposition of hemicelluloses is critically important for making cellulose accessible for degradation [75]. It is noteworthy that, an effective consortium of hemicellulose-degrading microbes is found in the termite gut [74]. Rouland et al. [75] reported that intrinsic hemicellulases have not been found, but they can be ingested with the feed in the fungus-feeding termites. In other termites, hemicellulose digestion seems to be produced with the aid of xylanases secreted by different symbiotic bacteria [75]. With many growing evidences in recent, the microbial decomposition of hemicellulose and cellulose components can continuously provide a variety of intermediate products that can be directly used by the host insects or further applied as a substrate for further fermentation reactions by the symbiotic yeasts. *C*. *pseudorhagii* is one of the unusual yeast species that can efficiently ferment D-xylose, suggesting a significant involvement for hemicellulose degradation processing in termite guts. Currently, the symbiotic yeasts with the ability to ferment xylose also have been found in association with other wood-ingesting beetles [25]. However, the mechanism of the association between yeasts and their hosts for these wood-feeding insects is largely unknown.

The symbiotic yeasts and their insect hosts are usually featured with a close association for their habitats. As more yeasts to be discovered from various habitats, the numbers of known yeast taxa have increased rapidly [25]. With a blast analysis, it has been showed that six yeast strains were actually reported to be linked with the wood-feeding insects, and all of which have been phylogenetically identified into the clade *Saccharomycotina* as illustrated in Fig 1. Two strains of *Candida* sp., BG01-7-21-025A-1-2^T^ and I-1 were isolated from Cerambycid beetle and a Japanese termite species, *Neotermes koshunensus*, respectively. The other four strains namely *Candida temnochilae* CBS 9938^T^, *C*. *temnochilae* CBS 9939^T^, *Yamadazyma akitaensis* NRRL Y-27710^T^, and *Pichia scolyti* SL-PY1^T^ were also beetle-associated yeasts. Thus, the yeast strain, *C*. *pseudorhagii* SSA-1542^T^, isolated from the wood-feeding termite, *R*. *chinensis*, may be potentially related to those yeasts isolated from some wood-feeding beetles due to a high rate of identity in terms of their pairwise sequences (79–88%). Clearly, these results suggest that the yeast symbionts isolated from a wood-feeding insect gut system may evolute some similar functions or characteristics aimed to assist their biomass processing.

### Conclusions

Lignocellulosic processes in xylophagous insects, such as wood-feeding termites, are considered to be a very efficient biodegradation reaction that is said to be largely due to a significant contribution from a variety of gut symbionts, including the yeasts. But, the diversity of those symbiotic yeasts residing in termite digestive tract and their functions or characteristics remains unclear for a long while. Our investigation suggests that the consortium of yeast symbionts hosted in guts of the wood-feeding termite, *R*. *Chinensis*, is particularly diversed with 92 strains from 18 yeast species, of which, seven were identified for new species and *C*. *pseudorhagii* SSA-1542^T^ was a common yeast strain presented in its gut system. Of these yeast symbionts to be identified, *C*. *pseudorhagii* SSA-1542^T^, along with other strains, has been confirmed with its unique and robust functions in xylanase production, and D-xylose fermentation for ethanol, which further suggests that some symbiotic yeasts in termite gut system may play an important role in hemicellulose degradation processing. Clearly, these unique yeasts may be potentially applied as a promising source of fermentation agents for biorefinery industry.

## Supporting information

S1 Fig Graphical abstract of xylanolytic and xylose-fermenting yeasts isolated from *Reticulitermes chinensis*. Representative schematic diagram of the screening and characterizing xylanolytic and xylose-fermenting yeasts isolated from the wood-feeding termite, *Reticulitermes chinensis* with focusing on the novel yeast species, *C*. *pseudorhagii* SSA-1542^T^, which showed the highest xylanase activity (1.73 and 0.98 U/mL with xylan or D-xylose substrate, respectively), ethanol yield (0.31 g/g), ethanol productivity (0.31 g/L·h), and its fermentation efficiency (60.7%) in 48 h.
(TIF)
