# Supplementary figures and images for: Screening and characterizing of xylanolytic and xylose-fermenting yeasts isolated from the wood-feeding termite, Reticulitermes chinensis
Source: PLoS One. 2017 Jul 13;12(7):e0181141. doi: 10.1371/journal.pone.0181141 (PMC5509302; doi:10.1371/journal.pone.0181141)

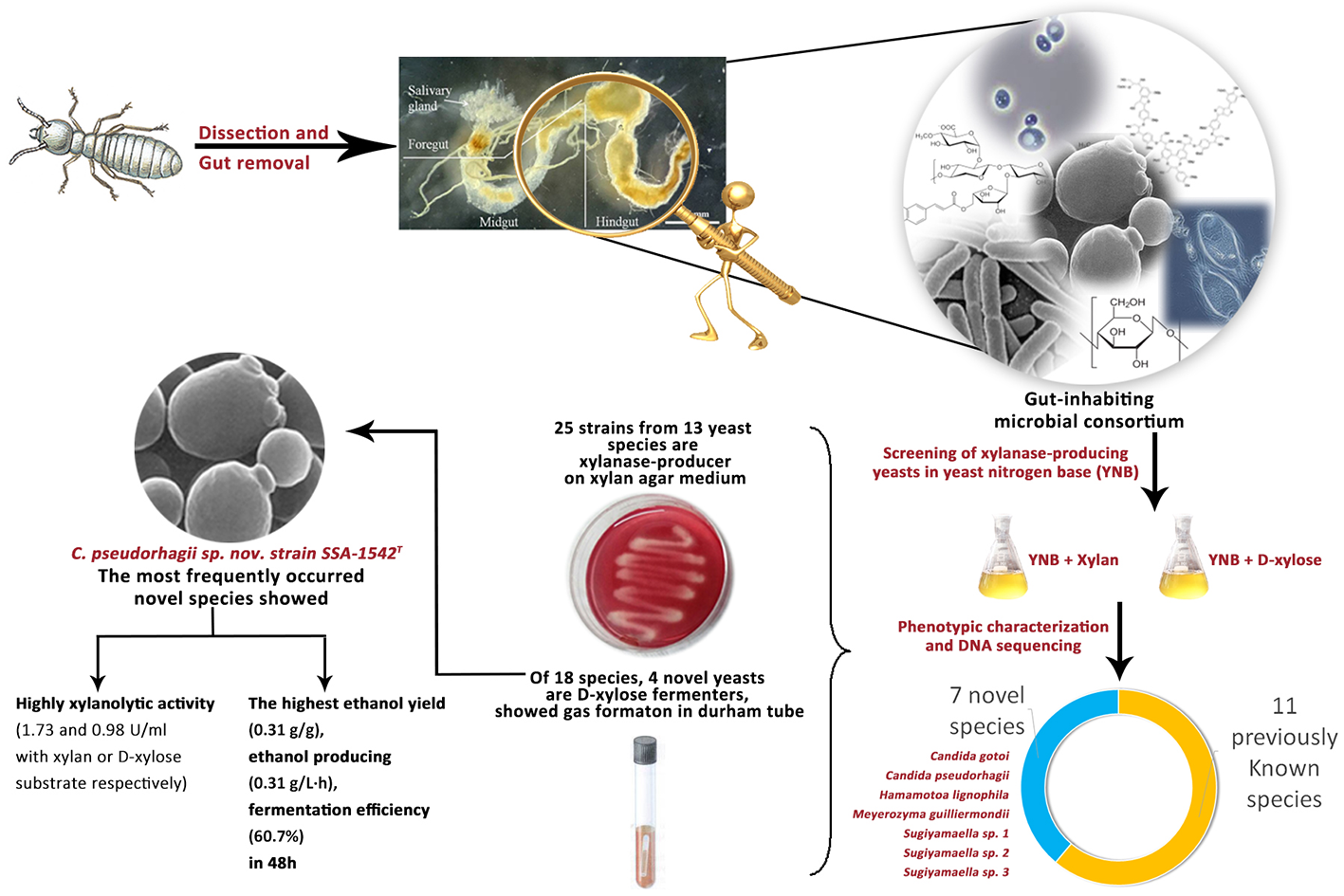

Supplement: S1 Fig — Representative schematic diagram of the screening and characterizing xylanolytic and xylose-fermenting yeasts isolated from the wood-feeding termite, Reticulitermes chinensis with focusing on the novel yeast species, C. pseudorhagii SSA-1542T, which showed the highest xylanase activity (1.73 and 0.98 U/mL with xylan or D-xylose substrate, respectively), ethanol yield (0.31 g/g), ethanol productivity (0.31 g/L·h), and its fermentation efficiency (60.7%) in 48 h. (TIF) [file pone.0181141.s001.tif]
